# Non-destructive inspection of food and technical oils by terahertz spectroscopy

**DOI:** 10.1038/s41598-018-36151-3

**Published:** 2018-12-21

**Authors:** Mindaugas Karaliūnas, Kinan E. Nasser, Andrzej Urbanowicz, Irmantas Kašalynas, Dalia Bražinskienė, Svajus Asadauskas, Gintaras Valušis

**Affiliations:** 1 Center for Physical Sciences and Technology, Saulėtekio Av. 3, 10257 Vilnius, Lithuania; 2 Physikalisches Institut, Goethe-University Frankfurt, Max-von-Laue Str. 1, 60438 Frankfurt am Main, Germany

## Abstract

Quality control and non-destructive monitoring are of notable interest of food and pharmaceutical industries. It relies on the ability of non-invasive inspection which can be employed for manufacturing process control. We hereby apply terahertz (THz) time-domain spectroscopy as non-destructive technique to monitor pure and degraded oils as well as hydrocarbon chemicals. Significant differences in the spectra of refractive index (RI) and absorption coefficient arising from the presence of ester linkages in the edible and technical oils were obtained. Explicit increase from 1.38 to 1.5 of the RI in all THz spectrum range was observed in hydrocarbons and mono-functional esters with the increase of molar mass. This fact is in contrast of RI dependence on molar mass in multi-functional esters, such as Adipate or vegetable oils, where it is around 1.54. Degradation products, Oleic Acid (OA) and water in particular, lead only to some changes in absorption coefficient and RI spectra of vegetable oils. We demonstrate that complex colloidal and supramolecular processes, such as dynamics of inverse micelles and oil hydrolysis, take part during oil degradation and are responsible for non-uniform dependence of optical properties on extent of degradation.

## Introduction

Terahertz (THz) imaging and spectroscopy is a well-known technique for detection and identification of concealed metal objects and chemical substances due to the unique properties of THz waves penetrate through most of the materials opaque to visible light, such as plastic, paper or cloths. On the other hand, a number of chemicals have fingerprint features in the THz range that can be exploited for identification via THz spectroscopy^1,2^. While the ability to screen the content of covered and sealed objects in a high speed is already used in security systems, the rapid and reliable detection and identification of substances are limited only to the pure chemicals, such as synthetic drugs or explosives^3–6^. The real-life applications have to deal with scattering and complexity of the medium containing chemicals under test^7,8^. Due to the fact that THz quantum energy is low and the radiation interacts only with the vibrational modes of the weakest bonds in the molecule, the THz range absorption lines are very sensitive to the small changes in the medium, i.e. the environment of the molecule. Therefore, the application of THz spectroscopy for food quality inspection is viable rather challenging^9–11^.

In the past decade, new emerging approach of THz spectroscopy – THz time-domain spectroscopy (TDS) in combination with chemometrics techniques in particular^6^ – showed a significant breakthrough in application for food quality inspection^12^. A number of studies has recently reported promising results that are addressed to the discrimination of transgenic crops^13,14^, early wheat grains germination detection^15^, the determination of tea types with the protected geographical indications^16^, as well as the discrimination between edible oils and used frying oils^17^. Recently, Yin *et al*. have demonstrated the possibility to determine edible oil type by working in the spectral range from 1.5 to 3.5 THz^18^. However, in the case of exploitation of “hidden” spectral features results of classification have to be considered very carefully. First, pure mathematical methods of spectral data analysis can overestimate the prediction results. Second, spectral features that can not be related to actual chemical processes that oils overcome during physical and chemical treatments, i.e. aging, wearing or mixing with additives, can not be accounted as reliable classification markers.

A substantial progress addressed to the oil investigation in THz range has been reported to date. Previously, it was shown that THz-TDS is a reliable technique for measuring optical properties of mineral oils^19^ as well as estimating the wear of lubricating oils^20^. Naftaly *et al*. reported the increase of refractive index (RI) with increasing the mineral oil viscosity and the decrease of absorption coefficient with increasing the mineral oil density, while the length of hydrocarbon chains has little effect on the optical properties^19^. In contrast, in the later study Naftaly and Miles claimed that RI might be related to hydrocarbon length as it influences the density and viscosity of the oil^20^. Other study by Gorenflo *et al*. has shown the ability to detect the contamination of lubricating polyglycol oils with water^21^. Gorenflo *et al*. used normalized integrated area under the RI and absorption coefficient curves to draw the dependence on water concentration in the lubricating oils as the oil spectra lack pronounced spectral features^21^. It was shown that the linear dependence of optical properties on water concentration and deeper analysis allowed to conclude that water forms the oil-water complexes like micelles rather than the water clusters. Nazarov *et al*. reported the absorption coefficient and RI in the range from 0.2 to 3 THz of chicken fat, sunflower oil and olive oil^22^. Jiusheng reported the RI and absorption coefficient spectra in THz frequency range for vegetable oils, namely sunflower, peanut, soybean, and rapeseed oils^23^. Jiusheng in his study attributed tentatively the spectral features of absorption spectra of vegetable oils in the range between 1.1 and 1.5 THz to the inter-molecular vibrations of hydrogen-bond bending in hydrocarbon chains^23^. A recent study by Dinovitser *et al*. showed the insufficient changes in the THz range absorption spectra of edible oils after the exposure to heat above smoke point temperature for 5 minutes^24^. The results of aforementioned reports are summarized in Table 1 and it shows a certain deviation of oils optical parameters even of the same type depending on the experimental set-ups and data analysis methods. In general, it is difficult to attribute observable changes in optical properties to actual microscopical differences of the oils, especially in natural ones with very diverse composition. Despite that fact, the non-destructive rapid THz-TDS technique is of a notable interest for oil inspection.

**Table 1:** Optical properties of various oils in THz spectral range at around 0.9 THz–*n* denotes RI, *α* denotes absorption coefficient and *f* denotes frequency.

Oils	*n*	*α*, cm^−1^	*f*, THz	Source
Mineral	1.47–1.5	0.7– > 10	0.03–2	Naftaly *et al*.^19^; Naftaly and Miles^20^
Synthetic	1.53–1.57	26–37	0.2–2	Gorenflo *et al*.^21^
Edible	1.54–1.55	4.7–5.4	0.2–3	Nazarov *et al*.^22^
Vegetable	1.48–1.49	7.3–8.5	0.2–1.5	Jiusheng^23^
Edible	n.a.	15*	0.2–1.3	Zhan *et al*.^17^
Vegetable	n.a.	2.5–20^+^	1.5–3.5	Yin *et al*.^18^
Vegetable	1.45–1.49	7–9	0.05–2	Dinovitser *et al*.^24^
Edible	1.51–1.53	7.5–12	0.1–1.6	this work
Lubricating	1.44–1.49	0.6–1.6	0.1–2	this work
Hydrocarbons	1.38–1.51	0.4– > 38	0.1–2	this work

*approximately estimated according the experimental description; ^+^at 1.5 THz frequency.

In this work, we enrich the family of the investigated edible oils including Hemp, Fish, Flax and Camelina oils, also few lubricating oils as well as several lighter hydrocarbon chemicals in the THz range using THz-TDS and determined the relation of optical properties to its chemical composition. It was discovered that the degradation process in edible oils manifests itself as non-uniform dependence of oil THz absorption spectra with increasing Oleic Acid (OA) concentration providing thus deeper insight into mechanisms behind. The results show complicated chemical processes of fatty acid (FA)-water complexes as inversed micelles governing the optical properties of oils above the critical micelle concentration (CMC). The chemical changes of heavily deteriorated oils lead to significant impact on the optical properties in the THz range that can be revealed using THz-TDS methods.

## Results

**Figure 1:**
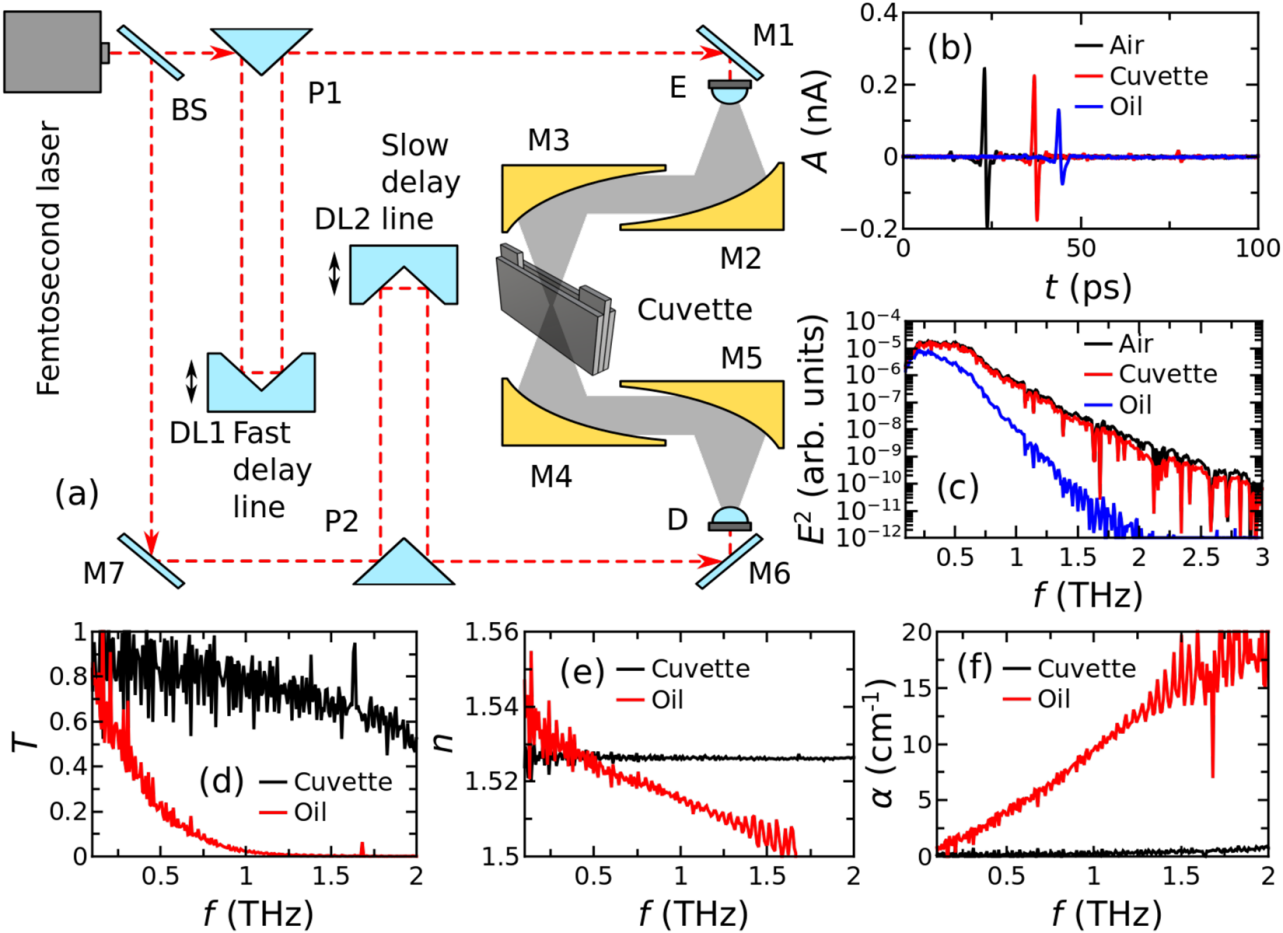
THz-TDS experimental set-up (**a**), transient waveforms of the THz radiation in time domain of reference, empty HDPE cuvette and unrefined Sunflower oil in the cuvette (**b**), corresponding THz pulses spectra in frequency domain (**c**), the transmission spectra *T* of the cuvette and oil (**d**), corresponding spectra of the RI denoted as *n* (**e**), and corresponding spectra of the absorption coefficient *α* (**f**).

The THz-TDS experiment was carried out under normal conditions (i.e. room temperature, air environment with relative humidity of 12%) using high density polyethylene (HDPE) cuvette as a oil holder in the spectrometer, Fig. 1a. Initially, the experimental set-up was carefully calibrated and its dispersion features were examined by measuring transient waveforms of the THz field propagated through free optical path, empty HDPE cuvette and unrefined Sunflower oil in the cuvette. Results are presented in Fig. 1b. The cuvette and oil causes pulse delay due to the RI change and amplitude decay due to the absorption in the optical path. In Fig. 1c, the dynamic range of the spectrometer is shown up to 3 THz, however when the oil is introduced in the cuvette, the spectral range decreases down to 2 THz owing to the substantial absorption of the oil in high frequency side. The corresponding spectra of transmission *T*, refractive index *n*, and absorption coefficient *α* of the cuvette and the oil are depicted in Fig. 1d–f, respectively. One can see that the cuvette has negligible dispersion and negligible absorption for THz radiation while the oil shows decreasing RI and increasing absorption towards higher frequency. Note that the sharp spectral lines at 1.64 and 1.68 THz are the contribution of the water vapor in air.

**Figure 2:**
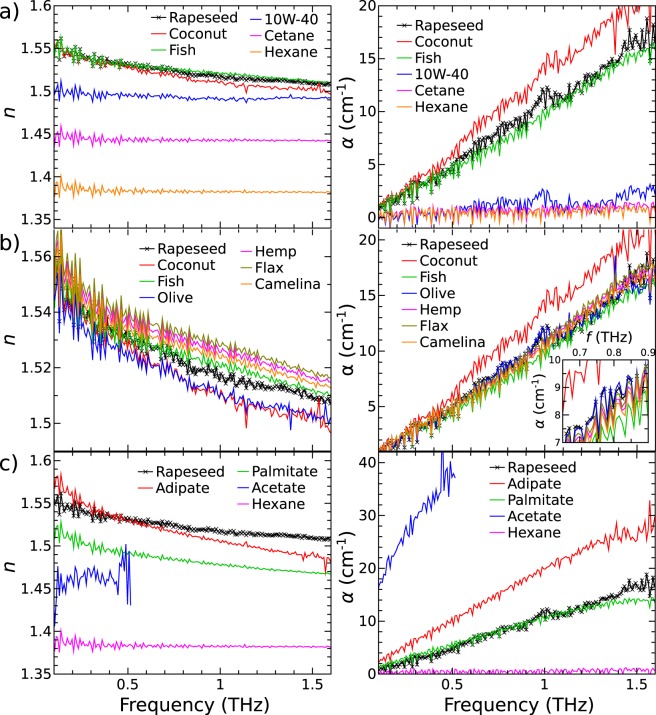
RI (left column) and absorption coefficient (right column) spectra of oils: (**a**) the most representative edible, fuel and lubricating oils; (**b**) refined and unrefined edible oils; (**c**) oil pairs of similar molar mass: Hexane ~ Acetate and Palmitate ~ Adipate. The inset in the right column panel (b) shows part of the plot in detail.

The RI and absorption coefficient spectra calculated from THz-TDS transient waveforms using equations (2) and (3) in Supplementary information (SI) for selected most representative edible and technical oils are depicted in Fig. 2. One can observe that hydrocarbon oils (10W-40, Cetane and Hexane) have somewhat lower RI and are more transparent to THz radiation in comparison to edible oils, Fig. 2a. The RI has no dispersion and it remains around 1.49, 1.44 and 1.38 for 10W-40, Cetane and Hexane, respectively, while it changes more rapidly from 1.54 to 1.51 towards higher frequency for edible oils. The uniform trend of the RI in the THz range suggests that oils do not seem to have specific absorption bands that could be attributed to any chemical features at room temperature. Nevertheless, some distinct differences can still be identified. The absorption coefficient of hydrocarbon oils is around 1.5 cm^−1^ while for vegetable oils and Fish oil it reaches around 10 cm^−1^, and in case of Coconut oil it is 12 cm^−1^ at 0.9 THz frequency. The absorption of edible oils increases linearly 3–4 times with increasing frequency. The steep increase of absorption coefficient towards the higher frequency side, up to 18 cm^−1^, can be attributed to the coupling of radiation into the acoustic phonon modes^20^.

Edible oils have similar molar mass due to the nature of fatty triglycerides, which are the main constituents of edible oils. These oils share very similar features in absorption and RI spectra, Fig. 2b. The spectra of refined oils, namely Rapeseed and Sunflower, were measured to be identical regardless of the different origin. All other unrefined vegetable oils, such as Hemp, Flax and Camelina, and Fish oil differ slightly in the optical properties by absolute values of RI and absorption coefficient due to a more diverse natural constituents. The absorption spectra of the Coconut oil is more like an exception among edible oils with higher absorption coefficient value and steeper increase with the frequency. Note, that Coconut oil is solid at room temperature, therefore its sample was melted by increasing the temperature above 24 °C. The results in Fig. 2b agree well with the ones reported before^19,20,23,24^. The small differences in optical properties of edible oils can be tentatively attributed to the different amounts of polar components. The THz spectra of all investigated edible oils together are available in SI Fig. 1. The results show small although distinguishable differences of RI while absorption coefficient spectra for unrefined and refined oils are rather identical with an exception of Coconut oil.

Lighter oils of mono-functional and di-functional esters have interesting trends of THz absorption and RI, Fig. 2c. Acetate has the most intensive absorption of all samples to such an extent that its spectra beyond 0.5 THz could not be measured. Even under lower frequencies the signal-to-noise ratio is small resulting in the significant data scattering. It can be explained by the fact that Acetate is a highly polar molecule. In contrast, Palmitate and Adipate behave much more like the rest of oils, showing similar trends of RI variation to those of vegetable oils, such as Rapeseed oil for example.

Considering the uncertainty of the measurement, the optimal thickness *d*_*opt*_ of the sample for THz-TDS experiment can be found as *d*_*opt*_ = 2/*α*^24^. Therefore, with the 4 mm oil thickness in our experiment, the best data reliability would be achieved for the frequency range at absorption coefficient of around 5 cm^−1^. As can be seen from Fig. 2 (also in Fig. 1e,f) the results with the lowest distortion are collected between 0.6 and 1.2 THz for edible oils. For hydrocarbon oils the data are more distorted, which can be explained by lower absorption that would require a thicker cuvette for optimal measurement. Another source of the noise to the signal is multiple reflections in the cuvette at the interfaces of different RIs which were not taken into account in data analysis for what it is necessary to introduce more sophisticated method of complex functions optimization^23^. Therefore, the accuracy of the measurement can be significantly increased for practical applications.

The degradation of the refined Rapeseed oil was simulated by introducing technical OA to imitate hydrolysis process. Since natural oils undergo hydrolysis quite easily, the tested sample of Rapeseed oil had some free FA even before adding OA. Therefore, free FA titration was performed with KOH solution in isopropanol, which established that 2.41 mg KOH was consumed per 1 g of refined Rapeseed oil, translating into 1.21% wt. of free FA such as OA. This titration was also performed on refined Sunflower oil, resulting in 2.39 mg KOH/g, hence 1.20% wt. of free FA. These amounts exceed slightly typical food-grade specification of 1% or less, however, the samples were prepared in normal conditions, leading to influx of ambient humidity, so such increase in acidity was not precisely controlled and is not particularly concerning.

**Figure 3:**
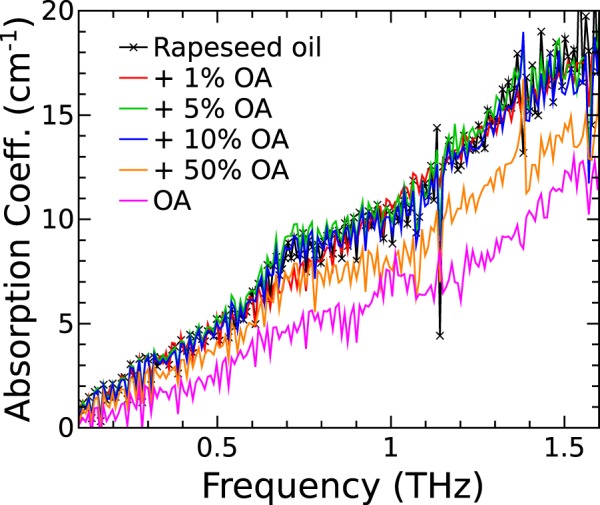
Absorption coefficient spectra of the refined Rapeseed oil (black line with symbols), OA itself (purple line) and different concentration mixtures of OA in Rapeseed oil.

The absorption coefficient spectra for refined Rapeseed oil and OA, as well as the mixtures of the two in various concentrations, are depicted in Fig. 3. The absorption spectra are rather difficult to separate at low OA concentrations. However, at higher concentrations the absorption coefficient decreases slightly with increasing concentration of OA. The only spectra that can be clearly resolved are that of OA itself and its 50% w/w mixture with Rapeseed oil. The RI spectra differ even less except for that of OA itself which is lower by absolute value (see SI Fig. 2). The reason for indistinguishable low concentration spectra is the non-uniform dependence of absorption coefficient on OA concentration that is going to be elucidated later. Note that, the absorption bands at 0.72 and 1.01 THz, that are correspondingly reflected in RI spectra, can be attributed tentatively to the intra-molecular vibrational modes^23^. The band remains at 0.72 THz independently on OA concentration, although, in case of pure OA sample, it appears at 1.01 THz. Therefore, without gradual transition it is difficult to link this spectral feature to the chemical processes in the oil.

**Figure 4:**
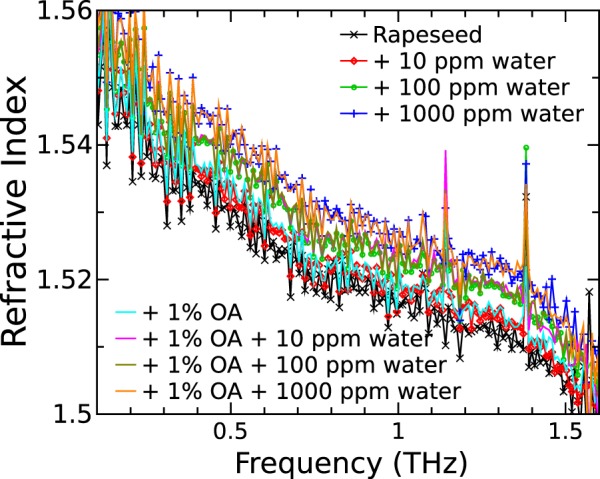
RI spectra of refined Rapeseed oil and refined Rapeseed oil with added 1% of OA at different water concentration levels.

The water contamination of refined Rapeseed oil was measured by adding a small amount of water at 10, 100, and 1000 ppm. The second sample set was taken with the same amount of added water and the 1% of added OA, which is usually present in natural oils^25^ and acts as surfactant^26,27^. The RI spectra of Rapeseed oil contaminated with water are shown in Fig. 4. One can see that RI spectra are slightly increasing with higher water concentration in case of Rapeseed oil with and without additional OA. The shape of RI spectrum remains unchanged. In contrast, the absorption spectra do not change with water concentration neither for Rapeseed oil nor the Rapeseed oil with additional OA (shown in SI Fig. 3). The sharp spectral lines at 1.14 and 1.38 THz can be attributed to water absorption, however there is no correlation between water concentration and absorption line depth. The same lines are visible in other spectra (see Figs 2 and 3). Therefore, it is likely to represent the absorption of the water vapor in atmosphere as the measurements were carried out at 12% relative humidity.

## Discussion

Experimental results show distinctly different RI values for several types of oils, Fig. 2. Even chemically simplest liquids, Hexane and Cetane, displayed a major difference in RI. This suggests clearly a correlation between molar mass and RI. As far as our knowledge goes, such correlation has not yet been reported in the literature. Therefore, more attention was directed to estimate the molar mass of tested materials. In order to explain major differences of optical properties in the THz range for technical and edible oils, low frequency RI at around 0.3 THz was calculated using relation (1) in SI for most representative hydrocarbons and esters and related to their molar mass.

Molecular weights of pure chemicals were readily available, while composition of the most refined vegetable oils had been analyzed in detail^28^. Their FA contents often appeared quite complex, however, many FA had similar molecular weights due to the same chainlengths of their hydrocarbon backbones. Therefore, FA of similar molecular weights were combined into several groups and average molar mass of key vegetable oils was calculated and is presented in SI Table 1. The molar mass of vegetable oils was estimated assuming that they contained 100% triglycerides, i.e. tri-functional esters of FA and glycerol. Therefore, some of the %mol concentrations, as retrieved from the article^28^ or material technical data sheets, had been normalized accordingly. However, unrefined oils might deviate from this assumption due to the presence of partial glycerides, free FA, phospholipids, sterols, vitamin E, waxes and other natural constituents. Nevertheless, the established values were sufficiently accurate to observe the correlative trends between RI and molar mass.

**Figure 5:**
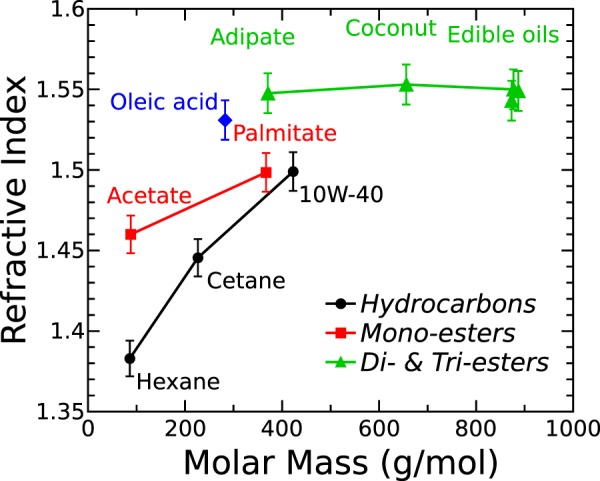
RI at around 0.3 THz as a function of molar mass of hydrocarbon and ester molecules. The error bars denote the 95% confidence interval.

The dependence of oils RI on the molar mass is depicted in Fig. 5. One can see a well-defined increase of RI with the molar mass, i.e. the length of the hydrocarbon chain, in case of hydrocarbons, and mono-functional esters as well. Like Hexane and Cetane, engine oil 10W-40 could also be considered as hydrocarbon, because the most abundant chemical compound in synthetic engine oils is a trimer of decene^29^. Therefore, the molar mass of 10W-40 should be similar to that of C_30_H_62_. When comparing 10W-40 to the other two hydrocarbons, its RI ~ 1.49 appears higher than 1.44 and 1.38 for Cetane (C_16_H_34_) and Hexane (C_6_H_14_), respectively (see Fig. 2a). A correlation between RI and molar mass of mono-functional esters is also clearly evident (see Fig. 2c). At 0.3 THz, Acetate recorded RI of 1.46 vs Palmitate RI of 1.50, molar mass of 88.1 vs 367 g/mol, respectively. In case of esters, some gradual RI reduction was evident with increasing frequency. Nevertheless, RI of Acetate remained clearly lower than that of its heavier homologue (i.e. Palimtate), despite of much higher absorption of the former.

The same trend of molar mass influence can be observed in vegetable oils, just the changes in RI were smaller, Fig. 2b. Coconut oil and Olive oil, being of lower molar mass than refined vegetable oils, recorded lower RI. To the best of our knowledge, no reports on THz spectra of Hemp, Fish, Flax and Camelina oils can be uncovered before this study. As it is seen from Fig. 2b, in agreement with expectations, their THz spectra did not show major differences from other natural oils, except Coconut and Olive. It must be noted that partial glycerides, free FA and other natural constituents might affect optical properties in the THz range.

Another major aspect is RI dependence on the functional groups in oils, Fig. 5. Molar mass of Hexane is very similar to that of Acetate (86.2 vs 88.1 g/mol, respectively), but their RI are very different, c.a. 1.38 vs 1.46. Molar mass values for Palmitate and Adipate are not far from identical at 367 and 371 g/mol. Their RI was recorded c.a. 1.50 and 1.55, respectively, which constituted a major difference, although both of them being esters. However, Adipate is a di-functional ester, while Palmitate is a mono-functional ester. Vegetable oils are composed nearly exclusively of tri-functional esters^25^. In many cases their RI were somewhat lower than that of Adipate despite of much higher molar mass. This implies that the influence in di-functional, tri-functional and quite likely multi-functional esters of molar mass on RI would not be evident. Therefore, additional functional ester groups play a significant role in the RI, especially in the case of large molecules. However, it is more difficult to correlate the spectra of unrefined natural oils to molar mass. Nevertheless, the relationship between RI and molar mass is very strong in hydrocarbon and mono-functional ester oils and must not be disregarded in future THz spectroscopy studies.

It might be speculative to provide possible explanations, why molar mass is so important for RI of mono-functional esters, and it appears insignificant for di-functional and tri-functional esters. However, it is worth noting that, while remaining liquid, multi-functional esters can engage into much broader variety of supramolecular transformations than mono-functional esters or hydrocarbons. Quite possibly, multi-functional esters have different mechanisms for phonon occurrence due to changes in their movement (bending, twisting, stretching etc.). Or else, due to much more pronounced amphiphilic properties, loosely-bound agglomerates, such as colloids, micelles, lamellas etc. can form, which might be able to act as high molar mass entities in their response to THz radiation^30^. Hydrogen bonding is much more prevalent in multi-functional esters, which might also have major consequences in phonon occurrence. A number of other hypotheses might also be brought up to explain such perplexing trends, but in any case much more experimentation and theoretical calculation is necessary to substantiate any of them. Nevertheless, this study for the first time demonstrates convincingly that variation of molar mass and the presence of ester linkages affects strongly RI in the THz range and even if the relationship is complicated the THz-TDS technique is viable to provide with such data.

**Figure 6:**
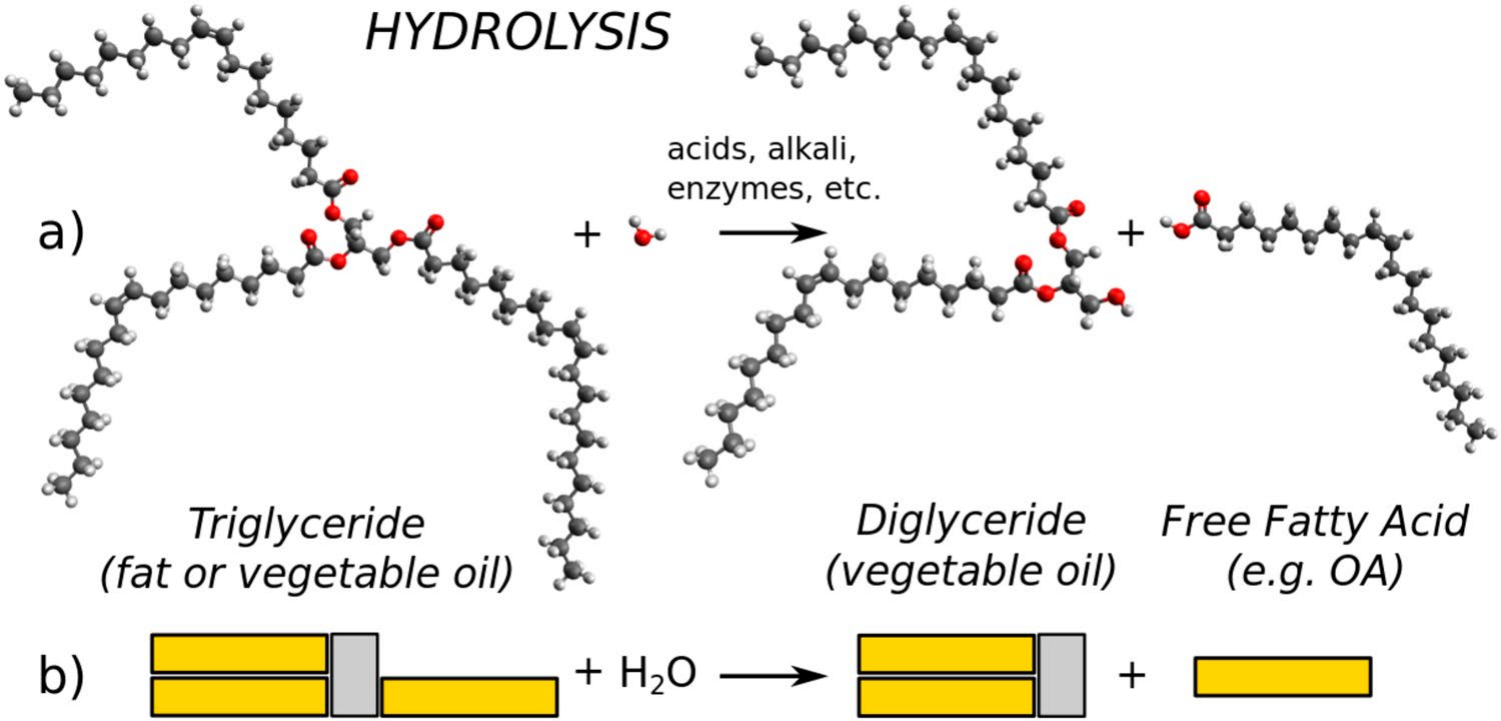
Schematic diagram of triglyceride break up through the hydrolysis process in reaction with water: the chemical picture in ball-and-stick model representation (**a**) and block diagram (**b**).

According to oleochemistry, vegetable oils deteriorate through two main processes^25,31–33^. One is oxidation that leads to the change of the number and location of the double bonds in triglycerides as well as formation of peroxides and other oxygenated degradation products. Oxidation affects severely nutritional properties of oils, therefore, it is often inhibited by adding antioxidants (e.g. vitamin E), avoiding free radicals (i.e. heat, light, metal surfaces, etc.) or restricting air access. Another process of deterioration is hydrolysis, when reacting with water the triglyceride molecules break down to diglycerides, monoglycerides or glycerol and release free FAs^34^. Simplified initial stage of hydrolysis is illustrated in Fig. 6 representing the chemical structures of OA and triolein (most abundant molecule in Rapeseed oil) with ball-and-stick model (a) and the block diagram (b). Since hydrolysis takes place easily in the presence of ambient humidity, typical standards for commercial edible oils require the concentration of free FA (such as OA) not to exceed 1%. As described above, refined Rapeseed and Sunflower oils were titrated, both showing acidities of 1.2%, which is a bit higher than specification limits. In contrast, the concentration of free FA in very deteriorated and contaminated vegetable oils can reach even 10% due to reactions with enzymes, metal ions, light, heat, etc.

**Figure 7:**
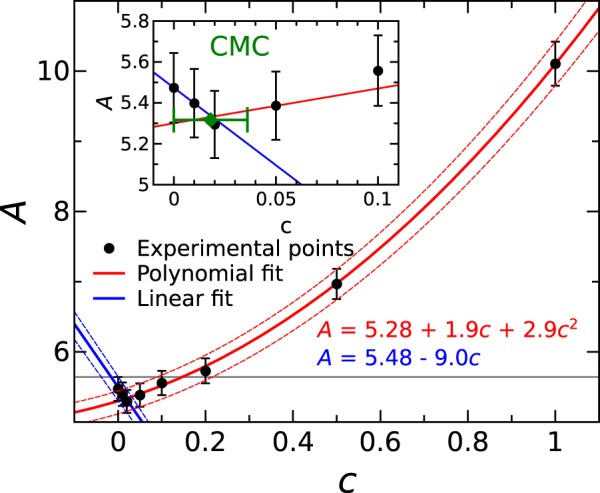
Dependence of integrated reciprocal absorption coefficient on OA concentration in refined Rapeseed oil. The dots are experimental points and lines are polynomial fits showing two intervals of the increasing absorption up to (3 ± 1.9)% of total free FA content followed by decrease with the increase of OA concentration. The gray horizontal line marks the probing limit at 16.7% defined by the initial part of the dependence including the uncertainty of the measurement. The inset shows the dependence interval in the vicinity of turning point and the estimated CMC (green diamond) at the 1.8%.

In order to analyze quantitatively the observed vertical shift of absorption spectrum with increasing OA concentration, the area under the absorption curve was considered as described with equation (4) in SI. In Fig. 7, the dimensionless integrated reciprocal of absorption coefficient *A* is depicted as a function of concentration of OA in the refined Rapeseed oil. The increase of integrated reciprocal absorption coefficient (decrease in absorption) with increasing concentration of OA was best fitted using second order polynomial with the coefficients *a*_0_ = 5.28 ± 0.03, *a*_1_ = 1.9 ± 0.2, and *a*_2_ = 2.9 ± 0.2. The initial decrease of integrated reciprocal absorption coefficient (increase in absorption) was fitted linearly using the coefficients *a*_0_ = 5.48 ± 0.01 and *a*_1_ = −9.0 ± 0.8. The intersection of the two fitting curves gives the concentration value of approximately 1.8% at which the dependence has its turning point. The non-uniform dependence of absorption on OA concentration explains the overlapped absorption spectra in Fig. 3. Indeed, there is an initial increase of absorption spectra followed by decrease when OA concentration is increase. To underline, the change of OA concentration in the oil can be unambiguously measured only above the limit of 15.6%. The observed phenomenon was measured repeatedly and the bars in Fig. 7 represent the standard error for the average of 5 measurements. The same trend was observed in RI, expressed through the dimensionless integrated optical thickness *B* calculated according to equation (5) in SI, dependence on OA concentration which is shown in the inset of SI Fig. 2, however, it is with greater uncertainty of the measurement. It must be noted that initial FA contents of 1.2% must also be accounted, therefore the turning point at 1.8% of added OA can be translated into (3 ± 1.9)% and the OA probing limit into 16.7% of total free FA contents.

**Figure 8:**
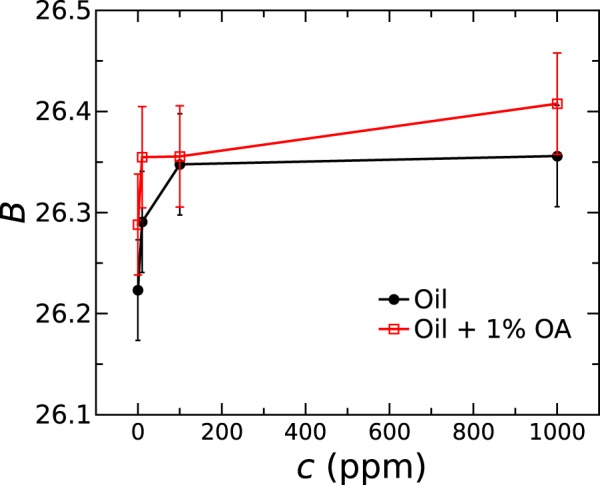
Integrated optical thickness *B* derived from RI spectra as a function of water concentration in refined Rapeseed oil (black line with closed circles) and in refined Rapeseed oil with 1% of added OA (red line with open squares).

In oil deterioration process not only the concentration of free FA but the concentration of H_2_O is important as well. Usually, the amount of water in oils under normal conditions is below 100 ppm. Although water-free oils can be purified reaching 1 ppm of H_2_O or less, such oil would absorb extensively water from the atmosphere. Typically, the THz radiation is strongly absorbed by water therefore one would expect to record even small changes of water concentration. The dependence of the integrated optical thickness *B* on water concentration is depicted in Fig. 8. It shows slight increase of RI with higher water concentration for oils with or without added OA. However, the standard error of the average of 5 measurements proves that in the oil degradation such small, but vital, water concentration changes are not explicitly reflected through the measurement of optical parameters using THz-TDS. The absorption coefficient spectra of Rapeseed oil do not change too much with increasing water concentration and the integral of reciprocal absorption coefficient *A* is depicted as a function of water concentration in SI Fig. 4 for oil as well as for oil with added OA. The results presented in Fig. 8 seem to contradict the fact that oil contamination with water at significantly greater scale is easily detectable^21^.

The above results can be elucidated in terms of oleochemistry. The FAs tend to form so called “inversed micelles” in oil with the hydrophilic end towards the center of sphere^26,27,35,36^. If the water is introduced, it is attracted into the center to form a core of inverse micelle. In this way the water dissolves in the oil only if certain amount of free FAs is present. Therefore, the micelles of OA form at certain critical concentration in oil whether water is present in oil or absent^35^. The experimentally observed turning point in absorption dependence on OA concentration (see Fig. 7) can be explained by formation of micelles within the concentration interval up to 5%. The results are in a good agreement with the work by Yan *et al*. where the CMC determination of micelle agglomeration using THz-TDS with high accuracy is demonstrated^37^. The turning point of absorption coefficient and RI dependence on surfactant concentration in the THz range is a clear signature of micellar transition. The quadratic dependence in the range of higher concentrations is overtaken by the optical properties of micellar agglomerates^37^. The experimental results of water-contaminated oil suggest that the water molecules are locked in the micelle cores^26,27^. The tightly bound FA-water colloids are formed. It is evident by the facts that the water absorption lines are independent on water concentration. The small change of RI could be explained by the change of micelle size. This deductive explanation is in a good agreement with the conclusions reached by Gorenflo *et al*. stating that water in the case of synthetic oils form the oil-water aggregates which behave like water in the inverse micelles^21^. They might form inverse micelles, which are different, resulting in less distinguishable CMC. All together, a great variety of complicated colloidal and supramolecular processes take part during oil degradation^30,34^. Therefore, THz spectroscopy techniques need more improvements to achieve higher accuracy, as might be required for identification of used oils. However, current THz methods are already sufficient to identify heavily deteriorated oils, such as highly degraded frying oils^17^ or overused engine oils^20^.

As mentioned, oil hydrolysis is not the only major mechanism of oil degradation that can be exploited for THz-TDS identification. The other direction of oil deterioration, i.e. oxidative degradation, proceeds very differently and involves much broader variety of chemical mechanisms: peroxide formation, oxidative scission, polymerization and others. In many cases oxidation products have much higher molar mass, e.g. oligomers of triglycerides. Since molar mass strongly affects THz spectra, influence of oxidative degradation needs particular attention in future studies. Another factor, which makes it difficult to accurately monitor the dynamics of inverse micelles in edible oils, is the presence of several molecular compounds, which can act as surfactants. Rapeseed oil also contains glycerides with linoleic and other FAs, in addition to those of OA. Extension of the approach, which is based on selecting specific compounds with similar molar mass, to more complex materials and their mixtures might lead to more findings, relating such compounds to their THz spectra. For example, study of mono- and di-glycerides solutions could provide with important information that could be crucial to construct the full picture of oil degradation process and its impact on optical properties in THz frequency range. Penetrating capabilities of THz are unique, so further improvement of the THz-TDS methods might produce major advancements in non-invasive testing and monitoring of oils, fuels, lubricants and many other related materials.

In conclusion, RI and absorption coefficient of selected edible and technical oils demonstrate discriminative features in the THz range that can be exploited for substance identification using THz-TDS. RI of hydrocarbon oils does not show dispersion in the THz range but in case of esters it displays a slight tendency to recede towards higher frequencies. Absorption coefficient around 10 cm^−1^ at 0.9 THz is greater for edible oils if compare to technical ones (around 1 cm^−1^), although for lighter hydrocarbon chemicals it increases with increasing polarity of smaller molecules up to 18 cm^−1^ for Adipate and more than 40 cm^−1^ for Acetate. RI correlates remarkably well with the molar mass of hydrocarbons and mono-functional esters increasing explicitly from 1.38 to 1.5, respectively. In di-functional ester (Adipate) and tri-functional esters (vegetable oils), the RI is even greater up to 1.54 ± 0.01, although with very little dependence on molar mass. The non-uniform dependence of oil THz absorption spectra on concentration of OA, which is the common degradation product in vegetable oils, was revealed. Above the OA CMC of (3 ± 1.9)% the dependence was best fitted using quadratic function. Around that concentration, the optical properties are affected by inverse micelle formation due to which the OA concentration in oil can be measured unambiguously only above 16.7%. The introduction of water up to 1000 ppm in the edible oil caused only slight RI increase without noticeable effects on absorption properties. The absence of absorption coefficient changes with increased water concentration in vegetable oils suggests that water is tightly bound in the inverse micelles. Complicated colloidal and supramolecular processes take part during oil degradation which was monitored via THz-TDS. It is a feasible technique for investigation of chemical processes in oils and identification of heavily deteriorated edible oils by measuring the extent of degradation non-invasively.

## Methods

Eleven regular commercially available from-the-shelf oils were selected to measure the optical properties in THz frequency range. Unrefined Sunflower (from *Rukola*, Lithuania), extra virgin Olive (from *Borges*, Spain), Coconut (from *Eko Pirk*, Lithuania), Hemp (from *Vitagra*, Lithuania), Flax (*Vitagra*), Camelina (*Vitagra*) oils, as well as refined Sunflower (*Rukola*) and refined Rapeseed (*Rukola*) oils were chosen for the edible vegetable oil group. Additionally, Fish oil was investigated (from *Orkla*, Norway). The FA composition of the edible oils used in experiment is listed in SI Table 1.

In addition to the edible oils, the optical properties of the semi-synthetic diesel motor oil 10W-40 (from *SCT Lubricants*, Germany) was measured. Other liquid hydrocarbons and esters were also tested. Hydrocarbons were represented by Hexane and Cetane (from *Sigma-Aldrich*), mono-functional esters by Acetate (i.e. ethyl acetate) and Palmitate (i.e. 2-ethyl hexyl palmitate as Estol 1543 from *Croda*, UK) and di-functional ester by Adipate (i.e. di-2-ethyl hexyl adipate as PlastHall DOA from *HallStar*, USA).

For the oil deterioration through hydrolysis experiment the refined Rapeseed oil with the concentration of free FAs less than 1.25% of total mass was diluted with the technical Oleic Acid (*Sigma-Aldrich*) at the fixed ratios from 1% to 50% w/w. The water contamination was investigated in refined Rapeseed oil adding 10, 100, and 1000 ppm of water to it. Second sample set was prepared by mixing refined Rapeseed oil with 1% of OA and adding 10, 100, and 1000 ppm of water to the mixture.

The experiments were carried out in the HDPE cuvette to assure necessary oil thickness for sufficient interaction length with the THz radiation. The cuvette was fabricated from two 4 mm thick HDPE slabs as front and rear windows separated with 4 mm thick HDPE frame in between. The distance between the front and rear walls was 4 mm determining the thickness of an oil layer in the cuvette. The total volume of the cuvette is 7 ml. The measured RI of the empty cuvette *n*_*cuv*_ is 1.528 without dispersion in the spectral range of interest (see Fig. 1e).

The spectra of selected edible and technical oils were measured using a commercially available THz time-domain spectrometer T-spec (from *Ekspla-Teravil*, Lithuania) in transmission mode. The THz-TDS experimental set-up is shown in Fig. 1a. A femtosecond laser with the central wavelength of 800 nm, less than 90 fs pulse duration and 80 MHz pulse repetition rate was used for THz pulse generation and detection. There are two optical paths for laser pulse created by the beam-splitter BS. One path is for THz generation at the photoconductive LT-GaAs emitter E and the other path is for THz pulse detection at the photoconductive LT-GaAs detector D. In the presence of the applied DC bias and the incident laser beam, an induced current is generated, which radiates a THz wave into the free space. Transient waveforms of the THz radiation were obtained by measuring photocurrent as a function of the time delay between the pump (THz pulse) and gate (laser beam) optical pulses. For more efficient collimation and focusing of THz radiation, a substrate lens fabricated from high resistance silicon was attached to the backside of each antenna. The fast delay line DL1 was used which is based on 10 times per second moving hollow retro-reflector with 120 ps time window what corresponds to ~8 GHz spectral resolution. A set of four parabolic mirrors M2–M5 was used to collimate, focus and collect THz radiation effectively. THz signal was detected by the digital signal processing card integrated into electronic module with analog-digital converter.

The spectral range of THz-TDS set-up in normal conditions is from 0.05 to 3 THz although for most of the oil samples actual dynamic range is from 0.1 to 1.6 THz. All measurements were carried out at room temperature and 12% relative humidity and typical THz spectra measured under such conditions are shown in Fig. 1c.

## Electronic supplementary material

Supplementary Information
