# Supplementary material for: Non-destructive inspection of food and technical oils by terahertz spectroscopy
Source: Sci Rep. 2018 Dec 21;8:18025. doi: 10.1038/s41598-018-36151-3 (PMC6303405; doi:10.1038/s41598-018-36151-3)
Supplement: Supplementary file 1 — Supplementary Information [file 41598_2018_36151_MOESM1_ESM.pdf]

Supplementary information for the paper: ´´Non-destructive inspection of food and technical oils by terahertz spectroscopy”

Mindaugas Karaliūnas[1,*], Kinan E. Nasser[2], Andrzej Urbanowicz[1], Irmantas Kašalynas[1], Dalia Bražinskienė[1], Svajus Asadauskas[1], and Gintaras Valušis[1]

[1]Center for Physical Sciences and Technology, Saulėtekio Av. 3, 10257 Vilnius, Lithuania
[2]Physikalisches Institut, Goethe-University Frankfurt, Max-von-Laue Str. 1, 60438 Frankfurt am Main, Germany
[*]mindaugas.karaliunas@ftmc.lt

Estimations of refractive index and absorption coefficient spectra

The time delay $\Delta t = t_s - t_r$ of the main THz pulse that have passed the sample of thickness d at the time t_s with respect to the reference pulse on the time t_r (see Fig. 1b in the main text) allows calculating the refractive index (RI)

$$n = 1 + \frac{c\Delta t}{d}, \tag{1}$$

where c is the speed of light in vacuum. The RI estimated using (1) corresponds roughly to the low frequency RI around 0.3 THz.

The fast Fourier transformation of the measured pulses in time domain gives the spectrum of THz field in frequency domain. The RI and absorption coefficient spectra can be calculated simultaneously from the measured phase and amplitude of the transmitted THz field through the sample $E_s(f)$ in respect to the incident field $E_{ref}(f)$. $n(f)$ of the sample was calculated as

$$n(f) = 1 - \frac{c\phi(f)}{2\pi f d}, \tag{2}$$

where $\phi(f)$ is the phase difference between $E_{ref}(f)$ and $E_s(f)$. The absorption coefficient $\alpha(f)$ was calculated as

$$\alpha(f) = -\frac{2}{d} ln \frac{|T(f)|}{L}, \tag{3}$$

where $T(f)$ is the transmission which is the ratio of the transmitted THz field through the sample $E_s(f)$ to the incident field $E_{ref}(f)$, $T(f) = E_s/E_{ref}$, and L is the loss of the signal at the interfaces. In the case of oil samples the interface air-cuvette-oil was taken into account

$$L = \frac{n(1+n_{cuv})^2}{(n+n_{cuv})^2}.$$

where n_{cuv} is the RI of the cuvette. Otherwise, only air-sample interface was taken into account

$$L = \frac{4n}{(1+n)^2}.$$

The examples of transmission, RI and absorption coefficient spectra of the empty cuvette, and unrefined Sunflower oil in the cuvette are shown in the panels d, e, and f of Fig 1 in the main text, respectively.

To evaluate small changes involving broad spectral contribution the area under the curve was calculated applying numerical integration. Moreover, if the integration of reciprocal absorption coefficient is used, the single number of dimensionless quantity can represent the whole absorption spectrum

$$A = k \int \frac{1}{\alpha(f)}\,\mathrm{d}f, \tag{4}$$

where k is conversion factor. If the optical thickness is used, the dimensionless numerical quantity can describe the RI spectrum

$$B = k \int dn(f)\, \mathrm{d}f. \tag{5}$$

The best result was achieved applying numerical integration in the range from 0.3 to 1.6 THz resulting the smallest uncertainty of 5 measurements. The error bars in the work correspond to the standard error of 5 measurements with the 95% confidence achieved using the coverage factor of 2.78.

Results

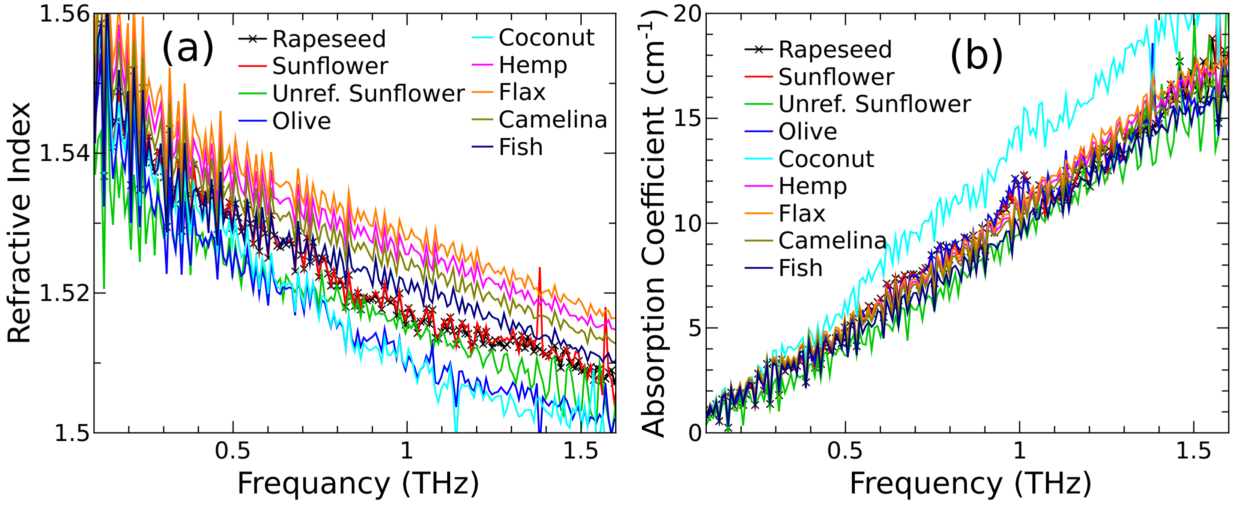

SI Figure 1. RI (a) and absorption coefficient (b) spectra of edible oils. The RI spectra are slightly different for unrefined oils in the order towards higher RI from Olive and Coconut, unrefined Sunflower, refined Rapeseed and Sunflower, Fish, Camelina, Hemp, and Flax. The absorption coefficient is less oil-type unique although 4 groups can be defined towards higher absorption: (1) unrefined Sunflower and Fish, (2) Camelina, Hemp, and Flax, (3) Olive, refined Rapeseed and Sunflower, and (4) Coconut oil. Note that best visual oil identification is obtained in vicinity of 0.9 THz frequency.

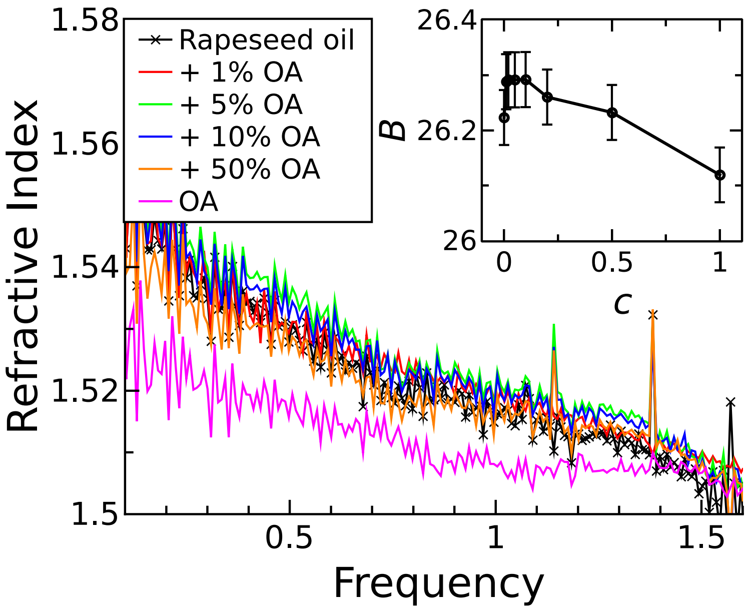

SI Figure 2. RI spectra of the refined Rapeseed oil (black line with symbols), OA itself (purple line) and different concentration mixtures of OA in Rapeseed oil. The refractive index slightely increases with initial increase of OA up to 5% and then decreases in the way that spectrum of Rapeseed oil wih 50% of OA overlapes the pure Rapeseed oil spectrum. The inset shows the integrated optical thickness B dependence on OA concentration with the error bars of 5 measurements.

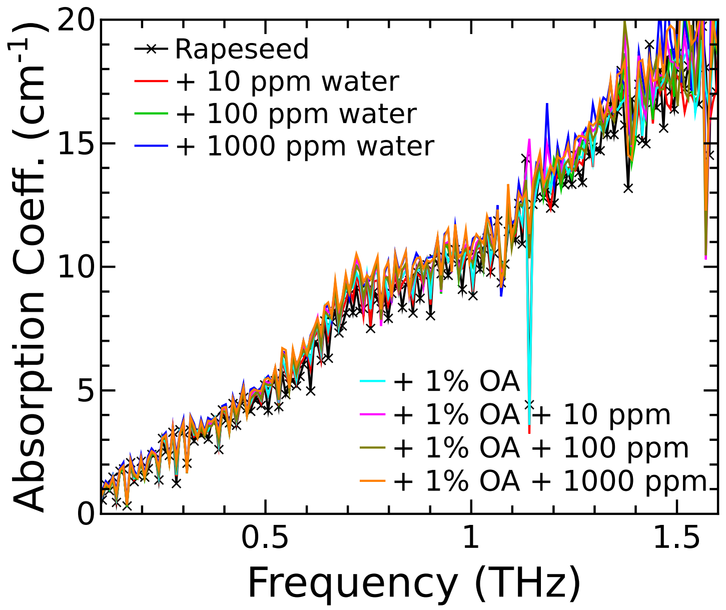

SI Figure 3. Spectra of absorption coefficient of refined Rapeseed oil (black line with symbols) and refined Rapeseed oil with added 1% of OA (cyan line) at different water concentration levels. In contrast to RI spectra, no observable changes can be seen in absorption spectra when water is added to the Rapeseed oil sample.

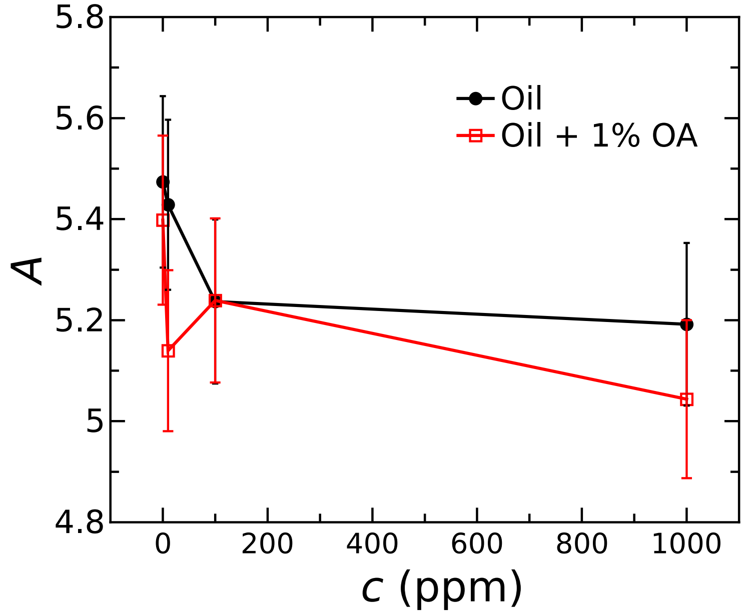

SI Figure 4. Integrated reciprocal absorption coefficient A as a function of water concentration in refined Rapeseed oil (black line with closed circles) and in refined Rapeseed oil with 1% of added OA (red line with open squares). In contrast to RI, the absorption coefficient does not show sufficient change to exceed the error of 5 measurements.

FA groups and chainlengths		Stearic acid	OA	FA of C18 poly unsat.	FA of C16	Myristic acid	FA of C6 to C12	FA of C20 to C22	Total molar mass, g/mol
Assumed individual molecular weights, g/mol		284.48	282.47	280	260	228.37	185	305	
Sample abbrev.	Sample name	Stearic acid, %mol	OA %mol	FA of C18 poly unsat., %mol	FA of C16, %mol	Myristic acid, %mol	FA of C6 to C12, %mol	FA of C20 to C22, %mol	
Rapeseed	Refined Rapeseed	1.7	63.5	20.8	4.8	0	0	9.2	887
Sunflower	Refined Sunflower	2.8	28	62.5	6.4	0.1	0	0.2	877
Olive	Unrefined Olive	2.2	62.4	17.1	17.4	0	0	0.9	873
Coconut	Unrefined Coconut	3	6.8	1.8	0	22	66.4	0	656
OA	Technical Oleic Acid	5	70	25					282
Hexane	Hexane								86.2
Cetane	Cetane								226
10W-40	Engine oil								423
Acetate	Ethyl acetate								88.1
Palmitate	Ethyl hexyl palmitate								367
Adipate	Di ethyl hexyl adipate								371

SI Table 1. Estimated average molar mass, g/mol, of key studied vegetable oils based on approximate conventional contents, %mol, of Fatty Acids (FA)[1,2] together with technical oils and hydrocarbons.

References

1. Fox, N. & Stachowiak, G. Vegetable oil-based lubricants – a review of oxidation. *Tribol. Int.* **40**, 1035 – 1046, DOI: https://doi.org/10.1016/j.triboint.2006.10.001 (2007).

2. Orsavova, J., Misurcova, L., Ambrozova, J. V., Vicha, R. & Mlcek, J. Fatty acids composition of vegetable oils and its contribution to dietary energy intake and dependence of cardiovascular mortality on dietary intake of fatty acids. *Int. J. Mol. Sci.* **16**, 12871–12890, DOI: https://doi.org/10.3390/ijms160612871 (2015).
